# Social Assistive Robots for Assisting Activity Professionals

**DOI:** 10.1093/geroni/igaa057.3406

**Published:** 2020-12-16

**Authors:** Jason Zamer, Anne Adams, Jenay Beer, Xian Wu, Jane Komsky

**Affiliations:** 1 SimpleC, LLC, Atlanta, Georgia, United States; 2 University of Georgia, Athens, Georgia, United States

## Abstract

Activity Professionals have high expectations for creating engaging and active resident social programming. A socially assistive robot (SAR) specifically designed for community-based settings has the potential to improve social programming. A SAR is suitable for engagement during times with social contact is restricted, such as COVID-19, other infectious outbreaks, weak immune system, or inability to move. We conducted an online survey to determine how a SAR can best support the responsibilities of Activity Professionals. Activity Professionals (N=19) completed the online questionnaire. Respondents (aged M=48.00, SD=12.87; 95% female, 100% native English speakers, 68% White/Caucasian, 21% Black/African American) were highly educated/experienced: 68% had a Bachelor’s degree or above, and 53% had 10-35 years of experience. Respondents worked in Independent Living (68%), Assisted Living (37%), Memory Care (26%), Skilled Nursing (21%), or Personal Care (11%). Respondents rated their job as very demanding (8 out of 10). Differences existed in terms of physical and temporal demands. Job satisfaction was high (average 8 out of 10; SD= 2). Respondents reported enjoyment in preparing, personalizing, and running activities. Least preferred was gathering residents for activities. Respondents wanted more help, but it depended on the task. Qualitative data analysis showed that help was desired for motivating residents to join activities, group communication, and resident devices. A SAR, equipped with the ability to reach every resident’s living quarter, has the potential to provide group communication, deliver engagement programs, and motivate residents to join events, providing Activity Professionals more time to engage with residents for more personal interaction.

